# L-shaped association between dietary zinc intake and the risk of developing cardiovascular disease in Chinese adults: A cohort study

**DOI:** 10.3389/fnut.2023.1032048

**Published:** 2023-03-17

**Authors:** Huanxiang Zhang, Shanjie Wang, Xia Gu, Hongbin Qiu, Yiying Zhang

**Affiliations:** ^1^Department of Epidemiology and Biostatistics, School of Public Health, Jiamusi University, Jiamusi, China; ^2^The first Affiliated Hospital of Jiamusi University, Jiamusi, China; ^3^Department of Cardiology, The Second Affiliated Hospital of Harbin Medical University, Harbin, China

**Keywords:** dietary zinc, cardiovascular, cohort study, adults, CHNS

## Abstract

**Background:**

Although the association of zinc (Zn) with cardiovascular disease (CVD) has been studied, no consensus has been reached on this relationship, particularly dietary Zn intake. The purpose of this study was to assess the effect of dietary Zn intake on the risk of CVD and to analyze whether this effect varied according to zinc consumption using representative data from China.

**Methods:**

11,470 adults from the China Health and Nutrition Survey (CHNS) were eventually enrolled. The dietary information was collected by the 3 day 24-h dietary recalls combined with dietary weighting method. CVD was defined as participants with self-reported physician-diagnosed apoplexy and/or myocardial infarction during the follow-up. Cox regression was used to calculate the hazard ratios (HRs) of CVD with 95% confidence intervals. Restricted cubic spline function plus Cox regression was used to visualize the influence trend of dietary Zn intake on new-onset CVD and to test whether this trend is linear. 2-segment Cox regression was established to address the nonlinear trend.

**Results:**

431 participants developed CVD, including 262 strokes and 197 myocardial infarctions. Compared with the lowest quintile (Q1), the adjusted hazard ratios and 95% confidence interval (CI) of CVD in Q2 to Q5 of dietary Zn intake were 0.72 (0.54, 0.97), 0.59 (0.42, 0.81), 0.50 (0.34, 0.72) and 0.44 (0.27, 0.71), respectively. The influence trend of dietary Zn intake on new-onset CVD was nonlinear and L-shaped. When dietary Zn intake <13.66 mg/day, increased dietary Zn intake was significantly associated with decreased risk of developing CVD (HR = 0.87, 95% CI: 0.82–0.92, *p*-value <0.0001).

**Conclusion:**

An L-shaped trend was observed between dietary Zn intake and the risk of developing CVD, indicating that dietary Zn intake should be improved moderately, but not excessively, for the benefit of cardiovascular disease.

## Introduction

Cardiovascular disease (CVD) refers to the assembly of diseases caused by the heart and blood vessels. Common CVD includes ischemic heart disease (myocardial infarction), ischemic stroke (apoplexy), arteriovenous disease and other conditions. Epidemiological evidence suggested that prevalent cases of CVD was 523 million, including 330 million in China (1, 2). According to the World Health Organization, 17.9 million people die of CVD each year, accounting for an estimated 32% of the total global deaths. 85% of cardiovascular deaths are due to heart attacks and strokes, and more than three quarters of cardiovascular deaths occur in developing countries. CVD remains the single largest cause of mortality worldwide and the leading cause of reduced quality of life (3). China has the highest cardiovascular mortality rate, and the disease burden due to CVD is increasingly severe and even higher than the global average (4). In addition, CVD has a strong genetic basis and is influenced by environmental factors. To date, the easiest way to prevent or reduce the occurrence of CVD is through changes in lifestyle factors, especially diet and exercise.

As an important cofactor of more than 2,000 transcription factors and more than 300 enzymes, zinc (Zn) is essential for regulating cell metabolism, proliferation and differentiation, as well as maintaining normal physiological functions of human body (5). Although foods such as lean meat, seafood and nuts are rich in zinc, about 17% of the world’s population is at risk of insufficient Zn intake (6, 7). Zn insufficient can lead to CVD, hypertension, diabetes, Alzheimer’s disease, and other chronic diseases (8–10). In recent decades, the role of Zn in CVD has become a research hotspot. Nevertheless, researchers have not come to an agreement on the effect of Zn, especially dietary Zn, on CVD (11). In addition, it is unclear whether different doses of dietary Zn intake have different effects on CVD.

As the most populous country in the world, it is particularly important to study the impact of Zn consumption on risk of CVD in China. However, to our knowledge, there is no study to analyze the relationship between dietary Zn intake and the incidence of CVD using large sample data in China. In order to figure out the relationship between dietary Zn intake and new-onset CVD in Chinese adults and analyze whether this relationship is affected by Zn intake, we conducted this study.

## Materials and methods

### Study population

**Figure 1:**
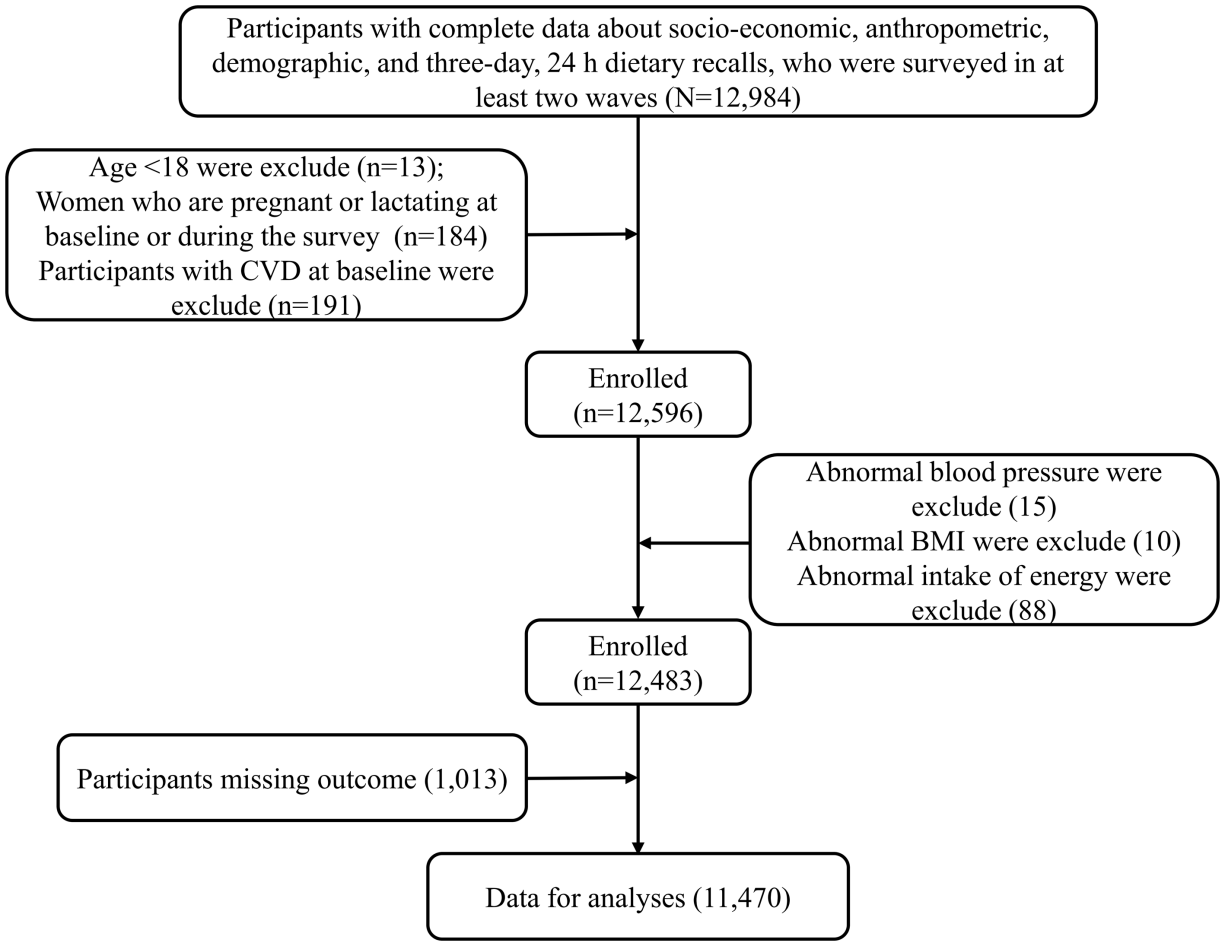
Study flow diagram.

The data for this study came from the China Health and Nutrition Survey (CHNS), a longitudinal follow-up survey based on community population. The survey included socio-economic status, health services, nutritional and dietary status. Please refer to relevant literature or official website^1^ for specific survey population and sampling methods (12). Since CHNS has provided more accurate dietary data since 2004 and can be obtained on the official website of CHNS, the subjects in this study were adults over 18 years old who participated in the survey from 2004 to 2015. Participants who have received at least two rounds of research surveys, and have complete sociodemographic indicators, socioeconomic indicators, lifestyle, anthropometric indicators, physical health status indicators and three consecutive 24-h diet recall data were considered valid subjects, and the first survey round was considered as baseline. The exclusion criteria include: (1) Age < 18, (2) Pregnant or lactating women, (3) Participants with CVD at baseline, (4) Exceeding the energy intake limit (Male: > 6,000 kcal or < 800 kcal, Female: > 4,000 or < 600 kcal), systolic blood pressure (SBP) < 40 mmHg or > 300 mmHg, diastolic blood pressure (DBP) < 30 mmHg or > 200 mmHg, body mass index (BMI) < 14 kg/ m^2^ or > 45 kg/ m^2^ or other unreliable observations. Finally, 11,470 participants were included in the final analysis (Figure 1). In addition, the number of people with complete data was 13,946 at baseline, so, the response rate was 11,470/13946 = 82.25%.

### Assessments of dietary intake

Three consecutive 24-h dietary recalls were used to assess dietary intake of participating individuals and household food consumption over the same 3-day period was calculated by subtracting the ending inventory from the starting inventory, combined weighing and measuring methods (the consumption of edible oil and condiments is obtained by the household food consumption). The accuracy of dietary data can be ensured by comparing each individual’s average daily dietary intake, calculated from household surveys with her dietary intake based on 24-h recall data. Food consumption data were converted into the nutrient intake and total energy using Chinese Food Composition Tables (FCTs). The cumulative average dietary intake from baseline to the latest year before the year of the first cardiovascular event or the end of follow-up was used for analysis, because it can better reflect long-term dietary intake and minimize within-person variation.

### Outcome identification

The outcome variable was whether CVD occurred during the follow-up period. The on-site investigators asked the participants whether they had myocardial infarction or stroke through interviews: “Has a doctor from a public hospital at or above the county level ever given you a diagnosis of myocardial infarction (no, yes, unknown) Or “Has a doctor from a public hospital at or above the county level ever given you a diagnosis of stroke (no, yes, unknown). New-onset CVD was defined as a self-reported physician diagnosis of myocardial infarction or stroke during follow-up and a certificate of stroke and/or myocardial infarction diagnosis should be provided. For participants who provided inconsistent answers during the follow-up, the first recorded stroke event was adopted to limit recall bias. The follow-up time was the date of the first discovery of CVD or the end of follow-up (2015) minus the date of first entering into the cohort.

### Assessments of covariates

The demographic, socioeconomic and lifestyle information of the participants was obtained through face-to-face interviews conducted by trained field investigators using well-designed questionnaires, including age, sex, race, marital status, residence, marital status, education level, occupational activity level, smoking status, alcohol intake. Weight and height were obtained by measuring to an accuracy of 0.1 cm in height and 0.1 kg in weight, after removing shoes and heavier clothing. Body mass index (BMI) was calculated by dividing body weight by the square of height (kg/m^2^). In a quiet environment, the participants sat quietly for more than 5 minutes. Then, after removing their heavy clothing, their blood pressure was measured three times, 10 min apart, by a trained health nurse using a calibrated mercury sphygmomanometer according to standard procedures, and the average of the three blood pressure measurements was used for the final analysis. Hypertension was defined as SBP ≥ 140 mmHg, DBP ≥ 90 mmHg, or taking antihypertensive medication according to the guidelines for preventing and treating hypertension in China (2010), or providing a self-report of diagnosis by a physician in public hospitals above the county level. Diabetes was defined based on self-report of diagnosis by a physician in public hospitals above the county level, or using oral medicine, injection of insulin to control blood glucose.

### Statistical analysis

SAS version 9.2 (SAS Institute, Cary, North Carolina, United States) and R version 4.0.5 were used for statistical analysis. Participants were categorized into quintile groups (< 7.87 mg/day, 7.87–9.63 mg/day, 9.63–11.38 mg/day, 11.38–13.56 mg/day, ≥ 13.56 mg/day) according to their Zn intake levels. Continuous variables that did not meet the normal distribution were represented by the median and interquartile range, while categorical values were expressed as numbers (percentage). Differences between groups were evaluated by using Kruskal–Wallis H test, and the chi-square test for the categorical variables.

Four Cox proportional hazards regression models were used to calculate the risk of CVD incidence with 95% confidence intervals. Model 1 was non-adjusted; Model 2 was adjusted for age, gender, race and energy intake. Model 3 was additionally adjusted for residence, marital status, education and activity level, smoking and drinking status, body mass index, hypertension and diabetes based on model 2; Model 4 was further adjusted for d intake of dietary fiber, niacin, vitamin C, vitamin E, calcium, iron, selenium, magnesium, copper, manganese based on model 3.

Restricted cubic spline (RCS) functions, with 4 knots, combined with Cox proportional hazards models were used to visualize the relationship between dietary Zn intake and the risk of developing CVD, after adjusting the variables of model 4. When non-linearity is detected, that the inflection point is calculated by the recursion algorithm, and a two-segment Cox proportional risk model was performed on both sides of the inflection point. Previous studies have observed associations between nutritional intake and health outcomes influenced by age, sex, BMI, smoking status, alcohol consumption, hypertension, and diabetes. Therefore, stratified analyses assessed whether these factors altered the association (13, 14). The test level was *α* = 0.05, two-tailed.

## Results

There was no collinearity between independent variables (Supplementary Table 1). A total of 11,470 adults subjects were eventually enrolled in this study, which consisted of 5,349 males and 6,121 females. Among the entire participants, the average Zn intake was 10.90 (SD, 3.91) mg/day, 40.44% had Zn intake lower than recommended nutrient intake (RNI, 12.50 mg/day for males and 7.50 mg/day for females aged 18 years and above), and 22.71% had Zn intake lower than estimated average requirement (EAR, 10.40 mg/day for males and 6.1 mg/day for females aged 18 years and above) (15). More details of Zn intake were shown in Table 1.

**Table 1:** Zinc intake among Chinese adults.

	Total	Male	Female
*N*	11,470	5,349	6,121
Dietary zinc intake (mg/d)	10.90 ± 3.91	11.86 ± 4.21	10.06 ± 3.43
Lower than RNI, no. (%)	4,638 (40.44)	3,299 (61.68)	1,339 (21.88)
Lower than EAR, no. (%)	2,605 (22.71)	2025 (37.86)	580 (9.48)

Mean ± SD for continuous variable and numbers (percentage) for categorical variables. RNI: Recommended Nutrient Intake (12.50 mg/day for male and 7.50 mg/day for female aged 18 years and above); EAR: Estimated Average Requirement (10.40 mg/day for male and 6.10 mg/day for female aged 18 years and above).

The baseline characteristics of the study population according to the intake of Zn were shown in Table 2. Participants with higher intake of dietary Zn were more likely to be younger, male, never married, smokers, and drinkers, had higher education levels, had a higher intake of energy, dietary fiber, niacin, vitamin C, vitamin E, calcium, iron, selenium, magnesium, copper, manganese, and less likely to be Han race, divorced, separated or widowed, light physically active, had lower education levels, had hypertension and diabetes. The result of comparing the indicators between non-CVD and new-onset CVD were shown in Supplementary Table 2.

**Table 2:** Baseline characteristics of participants according to quintiles of dietary zinc intake.

Characteristics	Zinc intake (mg/day)					
	*Q*1 (< 7.87)	*Q*2 (7.87–9.63)	*Q*3 (9.63–11.38)	*Q*4 (11.38–13.56)	*Q*5 (≥ 13.56)	*p*-value
*N*	2,301	2,288	2,289	2,298	2,294	
Male, no. (%)	693 (30.12)	894 (39.07)	1,003 (43.82)	1,251 (54.44)	1,508 (65.74)	<0.0001
Han race, no. (%)	2,148(93.35)	2038(89.07)	2031(88.73)	2001(87.08)	1,997 (87.05)	<0.0001
Urban, no. (%)	933 (40.55)	850 (37.15)	855 (37.35)	895 (38.95)	931 (40.58)	0.0312
Smoking, no. (%)	522 (22.69)	621 (27.14)	696 (30.41)	805 (35.03)	1,000 (43.59)	<0.0001
Drinking, no. (%)	500(21.73)	633(27.67)	693(30.28)	908(39.51)	1,084 (47.25)	<0.0001
hypertension, no. (%)	696 (30.25)	614 (26.84)	546 (23.85)	519 (22.58)	498 (21.71)	<0.0001
Diabetes, no. (%)	83 (3.61)	53 (2.32)	38 (1.66)	35 (1.52)	44 (1.92)	<0.0001
Marital status, no. (%)
Never married	112 (4.87)	127 (5.55)	127 (5.55)	144 (6.27)	178 (7.76)	<0.0001
Married	1,886 (81.96)	1,951 (85.27)	1,999 (87.33)	2,030 (88.34)	2,018 (87.97)	
Divorced, separated, widowed, etc	303 (13.17)	210 (9.18)	163 (7.12)	124 (5.40)	98 (4.27)	
Education level, no. (%)
≤ Primary school	1,057 (45.94)	946 (41.35)	885 (38.66)	791 (34.42)	693 (30.21)	<0.0001
Middle school	622 (27.03)	717 (31.34)	771 (33.68)	796 (34.64)	812 (35.40)	
≥ High school	622 (27.03)	625 (27.32)	633 (27.65)	711 (30.94)	789 (34.39)	
Activity level, no. (%)
Light	1,504 (65.36)	1,267 (55.38)	1,157 (50.55)	1,115 (48.52)	1,105 (48.17)	<0.0001
Middle	318 (13.82)	352 (15.38)	367 (16.03)	342 (14.88)	373 (16.26)	
Heavy	479 (20.82)	669 (29.24)	765 (33.42)	841 (36.6)	816 (35.57)	
Age (years)	53.00 (40.00, 64.00)	49.00 (38.00, 60.00)	47.00 (37.00, 57.00)	46.00 (36.00, 55.00)	46.00 (36.00, 55.00)	<0.0001
BMI (kg/m^2^)	23.15 (21.01, 25.79)	23.25 (20.95, 25.65)	22.77 (20.76, 25.32)	22.88 (20.82, 25.30)	23.02 (20.96, 25.31)	0.0010
Energy (kcal/day)	999.38 (810.35, 1,217.10)	1,237.48 (1,025.21, 1,484.10)	1,382.74 (1,168.33, 1,672.1)	1,571.34 (1,308.72, 1,896.30)	1,999.81 (1,617.42, 2,475.10)	<0.0001
Dietary fiber (g/ day)	7.04 (4.84, 9.64)	8.1 (5.73, 11.10)	8.44 (6.25, 11.35)	9.12 (6.89, 12.29)	11.16 (8.03, 16.27)	<0.0001
Niacin (mg/day)	7.24 (5.31, 9.47)	9.24 (7.27, 11.87)	11.03 (8.83, 13.78)	13.00 (10.62, 16.25)	17.71 (13.39, 22.62)	<0.0001
Vitamin C (mg/day)	46.00 (29.70, 68.30)	57.33 (38.02, 80.21)	65.77 (45.17, 93.17)	74.88 (51.60, 103.80)	86.55 (58.45, 122.27)	<0.0001
Vitamin E (mg/ day)	5.73 (3.78, 8.19)	7.11 (4.92, 10.06)	8.12 (5.47, 11.60)	8.99 (6.11, 12.80)	12.00 (7.87, 17.95)	<0.0001
Calcium (mg/ day)	210.97 (150.19, 284.95)	250.45 (184.89, 343.49)	287.2 (214.36, 393.40)	324.78 (244.01, 440.43)	408.23 (298.33, 583.59)	<0.0001
Iron (mg/ day)	12.63 (10.48, 14.75)	16.18 (14.29, 18.65)	18.74 (16.64, 21.49)	21.32 (19.11, 24.41)	27.05 (23.19, 32.72)	<0.0001
Selenium (mg/ day)	25.02 (17.17, 34.02)	28.76 (20.86, 39.88)	31.75 (23.65, 44.01)	36.20 (26.91, 49.50)	47.79 (33.51, 67.28)	0.0001
Magnesium (mg/day)	164.16 (129.63, 201.97)	201.33 (160.36, 247.70)	220.33 (177.22, 271.31)	245.54 (201.11, 302.69)	311.67 (240.89, 399.77)	<0.0001
Copper (mg/ day)	0.96 (0.72, 1.24)	1.25 (0.98, 1.54)	1.41 (1.12, 1.81)	1.65 (1.32, 2.10)	2.16 (1.64, 2.93)	<0.0001
Manganese (mg/ day)	2.91 (2.16, 3.67)	3.76 (2.98, 4.63)	4.28 (3.44, 5.26)	4.97 (3.94, 6.14)	6.25 (4.74, 8.31)	<0.0001

Median (q1, q3) for continuous variable and numbers (percentage) for categorical variables. BMI: body mass index.

431 participants developed new-onset CVD after 77,470 person-years of follow-up (mean follow-up time was 6.75 years) including 262 apoplexies and 197 myocardial infarctions. Table 3 showed that Zn intake had a negative impact on the incidence risk of CVD in the four Cox proportional hazards models. After controlling for age, gender, race, residence, marital status, education level, activity level, smoking status, drinking status, hypertension status, diabetes status, BMI, and the intake of energy, dietary fiber, niacin, vitamin C, vitamin E, calcium, iron, selenium, magnesium, copper, manganese, we found that the adjusted hazard ratios (HRs) and 95% confidence interval (CI) of CVD in Q2 to Q5 of the Zn intake were 0.72(0.54, 0.97), 0.59 (0.42, 0.81), 0.50 (0.34, 0.72) and 0.44 (0.27, 0.71), respectively, compared with Q1, and *p*-value for the trend was 0.0026. Similar results were obtained for apoplexy but not myocardial infarction. Furthermore, to avoid the influence of food sources on the relationship between single nutrition and disease, we also estimated the associations between Zn intake from animal and Zn intake from all other sources. Both Zn intake, Zn intake from meat and Zn intake from other sources were all associated with a reduced risk of CVD, as shown in Supplementary Table 3.

**Table 3:** HR (95% CI) for CVD, apoplexy, and myocardial infarction according to quintiles of dietary zinc intake.

	Zinc intake (mg/day)					*p*-value for trend
	*Q*1 (<7.87)	*Q*2 (7.87–9.63)	*Q*3 (9.63–11.38)	*Q*1 (< 7.87)	*Q*2 (7.87–9.63)	
CVD
Cases	102	97	82	71	79	
Incidence density	8.56	6.17	4.82	4.12	5.06	
Model 1 HR (95%CI)	1.00	0.71 (0.54, 0.93)	0.55 (0.40, 0.74)	0.47 (0.35, 0.64)	0.58 (0.43, 0.78)	< 0.0001
Model 2 HR (95%CI)	1.00	0.72 (0.54, 0.96)	0.58 (0.42, 0.78)	0.48 (0.34, 0.67)	0.39 (0.26, 0.58)	< 0.0001
Model 3 HR (95%CI)	1.00	0.73 (0.55, 0.97)	0.57 (0.42, 0.78)	0.47 (0.34, 0.66)	0.40 (0.27, 0. 06)	< 0.0001
Model 4 HR (95%CI)	1.00	0.72 (0.54, 0.97)	0.59 (0.42, 0.81)	0.50 (0.34, 0.72)	0.44 (0.27, 0.71)	0.0026
Apoplexy
Cases	61	60	49	39	53	
Incidence density	5.08	3.80	2.87	2.25	3.38	
Model 1 HR (95%CI)	1.00	0.74 (0.52, 1.05)	0.56 (0.38, 0.81)	0.44 (0.29, 0.65)	0.66 (0.45, 0.95)	0.0007
Model 2 HR (95%CI)	1.00	0.72 (0.50, 1.04)	0.54 (0.37, 0.81)	0.40 (0.26, 0.62)	0.39 (0.24, 0.64)	0.0003
Model 3 HR (95%CI)	1.00	0.74 (0.51, 1.07)	0.56 (0.37, 0.83)	0.41 (0.26, 0.63)	0.42 (0.25, 0.68)	0.0006
Model 4 HR (95%CI)	1.00	0.74 (0.51, 1.07)	0.57 (0.37, 0.86)	0.42 (0.26, 0.67)	0.44 (0.25, 0.80)	0.0079
Myocardial infarction
Cases	47	45	39	32	34	
Incidence density	3.90	2.85	2.28	1.85	2.16	
Model 1 HR (95%CI)	1.00	0.71 (0.47, 1.07)	0.57 (0.37, 0.87)	0.46 (0.29, 0.72)	0.54 (0.35, 0.84)	0.0051
Model 2 HR (95%CI)	1.00	0.78 (0.51, 1.19)	0.65 (0.41, 1.02)	0.53 (0.32, 0.87)	0.42 (0.23, 0.77)	0.0488
Model 3 HR (95%CI)	1.00	0.77 (0.51, 1.18)	0.62 (0.40, 0.98)	0.52 (0.32, 0.86)	0.42 (0.23, 0.77)	0.0402
Model 4 HR (95%CI)	1.00	0.78 (0.51, 1.20)	0.66 (0.41, 1.06)	0.57 (0.33, 0.99)	0.51 (0.25, 1.03)	0.3009

**Model 1:** non-adjusted. **Model 2:** adjusted for age, gender, race, and energy. **Model 3:** adjusted for age, gender, race, energy, residence, marital status, education level, activity level, smoking status, drinking status, BMI, hypertension, and diabetes. **Model 4:** adjusted for age, gender, race, energy intake, residence, marital status, education level, activity level, smoking status, drinking status, BMI, hypertension, diabetes, dietary fiber, niacin, vitamin C, vitamin E, calcium, iron, selenium, magnesium, copper, and manganese. CVD: cardiovascular disease; HR: hazard ratio; Incidence density: 1/1000 person-year.

Several sensitivity analyses were performed to assess the robustness of the relationship. Firstly, the results did not change substantially after further adjustments for the intake of cereals, potatoes, vegetables, fruits, nuts, meat, poultry, fish and shrimp, milk and eggs (Supplementary Table 4). Secondly, based on RNI, Zn intake was divided into two groups. The risk of CVD was reduced in the group with Zn intake ≥ RNI, which supported the above results (Supplementary Table 5). Thirdly, based on EAR, Zn intake was divided into two groups. The risk of CVD was reduced in the group with Zn intake ≥ EAR (Supplementary Table 6). Fourthly, to avoid the effects of dietary habits change, individuals with prior diabetes were excluded, and the results were consistent in the primary analysis (Supplementary Table 7). Fifthly, after imputation for missing values by multiple imputation, the associations remained consistent with previous results (Supplementary Table 8).

**Figure 2:**
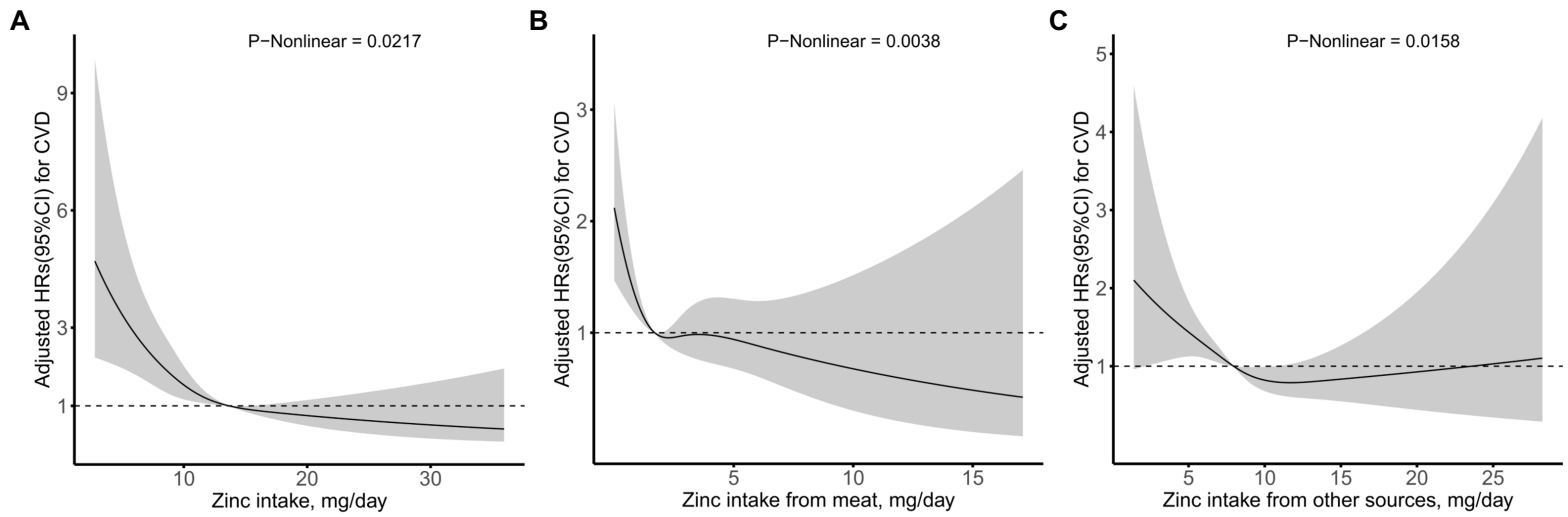
Relation of zinc intake from **(A)** total, **(B)** meat, and **(C)** other sources with risk of new-onset CVD*. *Adjusted for age, gender, race, energy intake, residence, marital status, education level, activity level, smoking status, drinking status, BMI, hypertension, diabetes, dietary fiber, niacin, vitamin C, vitamin E, calcium, iron, selenium, magnesium, copper, and manganese.

Fully adjusted Cox proportional hazards regression model combined with restricted cubic spline function, with 4 knots was conducted to clarify the relationship between the intake of dietary Zn, animal-derived-Zn, Zn from other sources and the risk of new-onset CVDs, and an L-shaped (*p*-value for nonlinearity was 0.0217, 0.0038, 0.0158, respectively) trend were found (Figure 2). A Cox proportional hazards model and a two-stage Cox proportional hazards model were used to evaluate the relationship between dietary Zn intake and the risk of new-onset CVD, respectively (Table 4). According to the results of the log likelihood ratio, it suggested that the Cox proportional risk model with 2-segment was more suitable to fit this relationship (*p*-value = 0.0150). Furthermore, we analyzed a threshold effect of Zn intake on the risk of new-onset CVD (Table 4). The inflection point of this L-shaped curve for dietary Zn intake was 13.66 mg/day. When dietary Zn intake <13.66 mg/day, the risk of developing CVD was significantly lower with the increment of Zn intake (HR = 0.87, 95% *CI*: 0.82–0.92, *p*-value <0.0001). But there was no significant association between Zn intake on the risk of new-onset CVD when dietary Zn intake ≥13.66 mg/day (HR = 0.96, 95% *CI*: 0.90–1.03, *p*-value = 0.2763).

**Table 4:** Threshold effect analyses of dietary zinc intake on the risk of new-onset CVD using 2-piecewise regression models.

Risk of developing CVD	Adjusted *HR* (95% *CI*), *P*-value
Fitting by the standard linear model	0.90 (0.85, 0.95), 0.0003
Fitting by the 2-piecewise linear model	
Inflection point	13.66
Dietary zinc intake <13.66 mg	0.87(0.82, 0.92), < 0.0001
Dietary zinc intake ≥13.66 mg	0.96 (0.90, 1.03), 0.2763
Log likelihood ratio	0.0150

Adjusted for age, gender, race, energy intake, residence, marital status, education level, activity level, smoking status, drinking status, BMI, hypertension, diabetes, dietary fiber, niacin, vitamin C, vitamin E, calcium, iron, selenium, magnesium, copper, and manganese.

**Figure 3:**
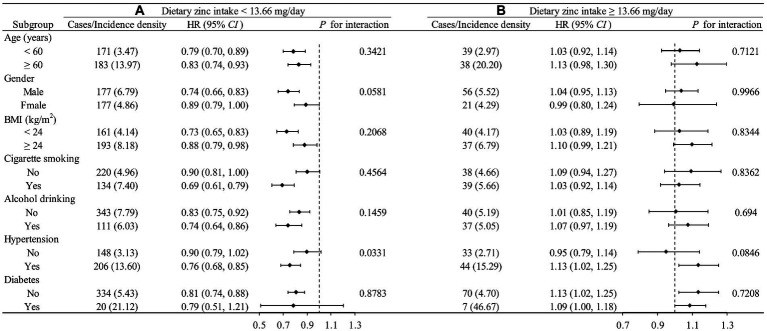
Stratified analysis by potential effect modifiers for the association between dietary zinc intake and new-onset CVD in various subgroups divided by 13.662 mg/day. **(A)** Dietary zinc intake <13.662 mg/day. **(B)** Dietary zinc intake ≥3.662 mg/day. Incident rate is presented as per 1,000 person-years of follow-up. Adjusted, if not stratified, age, gender, race, energy intake, residence, marital status, education level, activity level, smoking status, drinking status, BMI, hypertension, diabetes, dietary fiber, niacin, vitamin C, vitamin E, calcium, iron, selenium, magnesium, copper, and manganese; Incidence density: 1/1000 person-year.

We further performed a tentative analysis to assess whether there is any other potential factor that might affect the L-shaped relationship between the intake of the Zn and the newly diagnosed cardiovascular disease, as shown in Figure 3. The influence trends of dietary Zn intake on CVD remained similar in most subgroups by age, gender, BMI, cigarette smoking, alcohol drinking, and hypertension status. *p*-value for interaction for hypertension is 0.0331, suggesting that there is an interaction between Zn and hypertension. It is worth noting that the small number of diabetes cases may lead to non-clinical significance of the results in the diabetes subgroup.

## Discussion

Our study is the first relatively large-scale, retrospective cohort study to explore the effect of dietary Zn on new-onset CVD among Chinese adults. A nonlinear, L-shaped trend between dietary Zn and CVD, with an inflection point at about 13.66 mg/day was discovered. Higher dietary Zn intake, Zn intake from meat and Zn intake from other sources were all significantly associated with lower cardiovascular incidence, within a certain range. Both the sensitivity and stratified analyses illustrated that this relationship remained robust.

To our knowledge, the association between Zn and CVD in humans was first studied in 1988 (16). In this study, however, no association was found between regular use of Zn supplements and risk of developing CVD among people aged 65 years and older. Subsequent studies on dietary Zn intake and CVD have shown conflicting results.

Most of the previous studies have indicated that Zn plays a protective role in CVD. Dietary Zn has been found to be a protective nutrient against coronary artery disease in both Indians and Australians (17–19). According to the NHANES, after adjusting for covariates including demographics, comorbidities, renal function, and serum phosphorus levels, an increase in dietary Zn intake was independently associated with a lower probability of severe abdominal aortic calcification (AAC) (an increase of 1 mg daily dietary zinc intake was associated with an 8% lower probability of severe AAC) (20). Multiracial cohort studies of atherosclerosis supported this conclusion (7). The study found that a high-Zn diet was significantly associated with a lower risk of coronary artery calcification (CAC) progression in both men (HR = 0.697, 95%*CI*: 0.553–0.878; *p*-value = 0.002) and women (HR = 0.675, 95% *CI*: 0.496–0.919, *p*-value = 0.012, both groups were compared with the extreme group). In addition, the dietary intake of Zn increased by 1 mg, carotid artery intima-media thickness (cIMT) decreased by 3.36 μm (13). The risk of atherosclerosis, a fundamental process in CVD, has also been demonstrated to decrease with the increasing of Zn intake (21). In chronic kidney disease (CKD) patients, the beneficial effect of Zn intake or supplementation on cardiovascular disease risk factors remained significant (22). The dietary antioxidant index based on dietary Zn and other antioxidant nutrients was associated with decreased odds ratio of CVD (23). Furthermore, some studies demonstrated that higher dietary Zn intake is beneficial to reduce cardiovascular mortality (14, 24). In Iowa women, cardiovascular mortality decreased as dietary Zn intake increased (14). Compared with the lowest quintile Zn intake, the 2nd, 3rd, 4th, and 5th quintile Zn intake groups had a 39, 41, 43 and 63% lower risk of CVD death, respectively (*p*-value for trend = 0.07). When the effect of Zn supplementation on cardiovascular mortality was further investigated, no significant association was found. One possible explanation is that micronutrient supplementation may have a different effect than the same micronutrient in food. Some experts believe that Zn plays an important role in the maintaining cardiovascular health, and impaired zinc balance adversely affects cardiovascular dysfunction (25, 26).

Nevertheless, some literature supporting that the adverse effects of high Zn on CVD. For example, Milton et al. (27) concluded that in women, high dietary Zn intake may lead to higher CVD incidence rate, and Marcia et al. (28) found that Zn intake from red meat, but not from other sources, was associated with a higher risk of cardiovascular disease. Differences in study population, study outcomes, dietary Zn intake levels and sample size may contribute to the inconsistent conclusions. However, no significant association between Zn and CVD mortality was found in adults from Jiangsu, China (29). Compared with the first quartile of dietary Zn intake (mean: 10.4 mg/day), the adjusted *HR*s (95% *CI*) of CVD mortality in quartile 2 (mean: 11.7 mg/day), quartile 3 (mean: 12.3 mg/day), and quartile 4 (mean: 13.7 mg/day) were 0.97 (0.39, 2.42), 1.96 (0.91, 4.24), and 1.29 (0.51, 3.27), respectively. One possible explanation for the inconsistency with our results is that the average intake of Zn was higher than that we reported in our study, besides, different outcomes may play a role.

Our study provided a new idea for assessing the dose–response relationship between dietary Zn intake and the risk of developing CVD in the general population. The effect of dietary zinc intake on cardiovascular disease was observed in an L-shaped trend. The beneficial effect of increasing dietary Zn intake on CVD appears to peak in people with adequate Zn intake levels. That is, the risk of CVD decreased with increased dietary Zn consumption in those with a dietary Zn intake of <13.66 mg/day. Although the underlying mechanism of the inverse association between Zn intake and CVD remains to be studied. Based on the available evidence, our findings are biologically plausible. Firstly, high Zn levels decrease the expression of inflammatory factors. Zn supplementation can improve phosphate induced bone/cartilage transdifferentiation and vascular calcification of vascular smooth muscle cells (VSMCs) by inhibiting NF KB pathway (30). After 6 months of Zn supplementation in the intervention group, compared with placebo, the plasma Zn concentration in the intervention group was significantly increased, while the concentration of inflammatory related factors such as high-sensitivity C-reactive protein (hsCRP) was significantly decreased, reported by a clinical randomized placebo-controlled trial (31). The team further conducted cell experiment and the results supported the conclusions of the above tests (31). Secondly, Zn is a cofactor of copper Zn superoxide dismutase and participates in the regulation of multiple antioxidant enzymes. Impaired superoxide dismutase function causes oxidative stress (32). Thirdly, NO has the functions of inhibiting platelet aggregation, inhibiting smooth muscle cell (SMC) proliferation and inhibiting endothelial cell apoptosis. Zn can affect the pathogenesis of CVD by regulating the production and release of NO (33). Fourthly, Zn is key to the proper removal of reactive oxygen species and nitrogen (34). Therefore, Zn deficiency will increase the production of reactive oxygen species, promote apoptosis of endothelial cells and vascular smooth muscle cells, activate proinflammatory cytokines, and amplifie the oxidation of low-density lipoproteins, thereby exacerbating oxidative stress and ultimately leading to CVDs (35–37). Fifthly, *in vitro* experiments, endothelial cells cultured in Zn-deficient medium lead to decreased integrity and increased permeability of endothelial cells, and Zn deficiency also affects the severity of apoptosis. In turn, Zn-rich media improved their structure (38, 39). Sixthly, Proteomic studies have shown that the phenotype of VSMCS in large arteries may be altered in animal models of Zn deficiency, which may lead to vascular diseases (40). Studies of mice fed a Zn-deficient diet found that inadequate Zn intake increased lipoprotein concentrations, increased remodeling of VSMCS, increased inflammation, and induced atherosclerotic plaque formation (34, 41).

Whereas, the beneficial effect of Zn intake on CVD became statistically insignificant, when dietary Zn intake ≥13.66 mg/day. Ting Yin et al. also found that there was an L-shaped association between Zn and the prevalence of CVD risk in American adults, but the inflection point of Zn was 6.61 mg/day, while our study showed that the inflection point of Zn was 13.66, which was closer to the high recommended intake of zinc (12.5 mg) (42). One possible explanation for the different inflection points of dietary Zn was the difference between the two studies (cross-sectional study vs. cohort study). Another possible explanation could be attributed in part to differences in the sources of zinc between the two studies. Ting Yin et al. studied a Western population with an animal-based diet, while we studied an Asian population with a plant-based diet. The animal source of Zn had higher bioavailability than plant source because of the possible presence of phytate, which inhibits Zn absorption in the intestine (43). In addition, higher intake of meat including processed meat, unprocessed red meat, or poultry was significantly associated with increased risk of incident CVD (44). Therefore, it seems reasonable that the inflection point of the relationship between zinc and CVD in the Western population with a diet dominated by animals is lower than that in the Chinese population with high phytic acid content of corn, cereals, rice, legumes and other foods as the staple food (45).

Laura M Pompano and Erick Boy reviewed 27 articles and concluded that compared with high-dose and short-term Zn supplementation, low-dose and long-term Zn supplementation improved more cardiovascular risk factors (46). One plausible explanation is that the body’s absorption of Zn is limited. When the intake of Zn exceeds the body’s absorption capacity, the protective effect of Zn on CVD will not be further enhanced even if the intake of Zn is increased. Besides, although Zn has antioxidant and anti-inflammatory effects, too high plasma Zn concentration can have the opposite effect, such as inhibiting lymphocyte function and causing abnormal expression of proinflammatory cytokines (47). Excessive intake of Zn leads to elevated systemic blood pressure through oxidative stress, and it has been reported that excessive clearance of ROS or RNS and their derivatives by antioxidant supplementation may disrupt cellular signaling pathways and may increase the risk of chronic disease (48, 49).

Several limitations need to be acknowledged. Firstly, although the dietary assessment was a 24-h dietary recall and was conducted in every survey wave (usually 2 years), which only reflects the short-term dietary situation, and the changes in nutritional intake during different seasons of the year may be overlooked. The accuracy of 24-h dietary recall method for assessing nutrition intake has been verified and the average intake over 3 days can offer a relatively valid estimate of usual diet, as has been shown in earlier research using the CHNS (50–52). In addition, the method of cumulative dietary nutrient intake was adopted to minimize dietary measurement errors and minimize within-individual variation. Therefore, it may not cause fatal to the significance of the study. Secondly, there is a lack of information on Zn supplementation in this survey. Whereas the proportion of Chinese individuals using dietary supplements was quite low, therefore, we speculated that our results may not change substantially due to the use of dietary supplements. Thirdly, apoplexy and myocardial infarction cases in this database are based on self-reports, and only these two diseases were used to define CVD in this study.

To sum up, this is the first research revealed an L-shaped trend between dietary Zn intake and the risk of developing CVD in Chinese adults. That is, increasing dietary Zn intake significantly reduced the risk of developing CVD in subjects with dietary Zn < 13.66 mg/day, and the beneficial effect of Zn on CVD became insignificant in the presence of dietary Zn ≥ 13.66 mg/day. These findings indicated that raising dietary zinc to a certain level can help reduce the risk of cerebrovascular diseases, especially stroke, which not only provides a theoretical basis for the prevention of cardiovascular diseases but also emphasizes the importance of adequate, but not excessive, dietary zinc intake.

## Data availability statement

The datasets presented in this study can be found in online repositories. The names of the repository/repositories and accession number(s) can be found at: https://www.cpc.unc.edu/projects/china.

## Ethics statement

The studies involving human participants were reviewed and approved by the Institutional Review Boards of the National Institute of Nutrition and Food Safety of China (Beijing) and the University of North Carolina (Chapel Hill, NC, United States). The patients/participants provided their written informed consent to participate in this study.

## Author contributions

HQ, YZ, and HZ contributed to the design and conduct of the research. HZ and SW carried out data analysis and the initial draft of the paper. XG and YZ conducted the data collection and advised on statistical analysis. All authors reviewed and edited the draft and approved the final version of the manuscript.

## Funding

This research was funded by the Natural Science Foundation of Heilongjiang Province of China (ZD2022H006), Postdoctoral Science Foundation of Heilongjiang Province of China (LBH-Q21047), Research Innovation Team of Metabolic Disease Prevention and Treatment, the first affiliated hospital of Jiamusi University (202303), Scientific and Technological Innovation Team of Jiamusi University (cxtd202101), North Medicine and Functional Food Characteristic Subject Project in Heilongjiang Province (No. HLJTSXK-2022-03), and Gout Etiology and Functional Food Research Innovation Team.

## Conflict of interest

The authors declare that the research was conducted in the absence of any commercial or financial relationships that could be construed as a potential conflict of interest.

## Publisher’s note

All claims expressed in this article are solely those of the authors and do not necessarily represent those of their affiliated organizations, or those of the publisher, the editors and the reviewers. Any product that may be evaluated in this article, or claim that may be made by its manufacturer, is not guaranteed or endorsed by the publisher.

## Footnotes

^1^ http://www.cpc.unc.edu/projects/china
