# Supplementary material for: L-shaped association between dietary zinc intake and the risk of developing cardiovascular disease in Chinese adults: A cohort study
Source: Front Nutr. 2023 Mar 17;10:1032048. doi: 10.3389/fnut.2023.1032048 (PMC10064069; doi:10.3389/fnut.2023.1032048)
Supplement: Supplementary file 1 [file Data_Sheet_1.docx]

Supplementary Table 1. Collinearity analysis

|  |  | Total | |  | Mele | |  | Female | |  | Non-CVD | |  | CVD | |
| --- | --- | --- | --- | --- | --- | --- | --- | --- | --- | --- | --- | --- | --- | --- | --- |
| Variable | Types of variables | TOL | VIF |  | TOL | VIF |  | TOL | VIF |  | TOL | VIF |  | TOL | VIF |
| Intercept | - | - | 0.00 |  | - | 0.00 |  | - | 0.00 |  | - | 0.00 |  | - | 0.00 |
| Gender | Categorical variable | 0.47 | 2.11 |  | - | - |  | - | - |  | 0.47 | 2.13 |  | 0.50 | 2.00 |
| Race | Categorical variable | 0.94 | 1.07 |  | 0.93 | 1.08 |  | 0.94 | 1.07 |  | 0.94 | 1.07 |  | 0.90 | 1.12 |
| Residence | Categorical variable | 0.79 | 1.27 |  | 0.8 | 1.25 |  | 0.77 | 1.30 |  | 0.79 | 1.27 |  | 0.75 | 1.34 |
| Smoking status | Categorical variable | 0.56 | 1.79 |  | 0.93 | 1.07 |  | 0.96 | 1.04 |  | 0.55 | 1.81 |  | 0.61 | 1.63 |
| Drinking status | Categorical variable | 0.67 | 1.48 |  | 0.92 | 1.09 |  | 0.96 | 1.04 |  | 0.67 | 1.48 |  | 0.64 | 1.56 |
| Hypertension | Categorical variable | 0.82 | 1.22 |  | 0.85 | 1.18 |  | 0.79 | 1.26 |  | 0.83 | 1.20 |  | 0.80 | 1.24 |
| Diabetes | Categorical variable | 0.97 | 1.04 |  | 0.97 | 1.03 |  | 0.96 | 1.04 |  | 0.97 | 1.03 |  | 0.90 | 1.11 |
| Marital status | Categorical variable | 0.79 | 1.26 |  | 0.82 | 1.22 |  | 0.78 | 1.27 |  | 0.79 | 1.26 |  | 0.83 | 1.21 |
| Education level | Categorical variable | 0.67 | 1.49 |  | 0.72 | 1.39 |  | 0.64 | 1.56 |  | 0.67 | 1.5 |  | 0.74 | 1.34 |
| Activity level | Categorical variable | 0.68 | 1.47 |  | 0.68 | 1.48 |  | 0.69 | 1.44 |  | 0.68 | 1.47 |  | 0.58 | 1.73 |
| Age | Continuous variables | 0.60 | 1.66 |  | 0.63 | 1.58 |  | 0.56 | 1.78 |  | 0.61 | 1.64 |  | 0.57 | 1.74 |
| BMI | Categorical variable | 0.91 | 1.10 |  | 0.87 | 1.15 |  | 0.91 | 1.10 |  | 0.91 | 1.10 |  | 0.81 | 1.24 |
| Energy intake | Continuous variables | 0.25 | 3.97 |  | 0.27 | 3.69 |  | 0.23 | 4.35 |  | 0.25 | 3.95 |  | 0.17 | 5.83 |
| Diet-fiber intake | Continuous variables | 0.33 | 3.07 |  | 0.27 | 3.70 |  | 0.40 | 2.51 |  | 0.33 | 3.04 |  | 0.20 | 4.94 |
| Niacin-intake | Continuous variables | 0.30 | 3.32 |  | 0.29 | 3.47 |  | 0.33 | 3.05 |  | 0.30 | 3.34 |  | 0.29 | 3.49 |
| Vitamin C intake | Continuous variables | 0.59 | 1.71 |  | 0.61 | 1.63 |  | 0.55 | 1.82 |  | 0.59 | 1.71 |  | 0.50 | 2.01 |
| Vitamin E intake | Continuous variables | 0.49 | 2.06 |  | 0.46 | 2.18 |  | 0.52 | 1.94 |  | 0.49 | 2.06 |  | 0.45 | 2.21 |
| Calcium intake | Continuous variables | 0.25 | 3.94 |  | 0.20 | 4.88 |  | 0.32 | 3.12 |  | 0.25 | 3.96 |  | 0.26 | 3.88 |
| Iron intake | Continuous variables | 0.35 | 2.84 |  | 0.29 | 3.39 |  | 0.43 | 2.31 |  | 0.35 | 2.86 |  | 0.31 | 3.26 |
| Selenium intake | Continuous variables | 0.32 | 3.08 |  | 0.29 | 3.43 |  | 0.38 | 2.64 |  | 0.32 | 3.10 |  | 0.33 | 3.06 |
| Magnesium intake | Continuous variables | 0.13 | 7.92 |  | 0.11 | 8.92 |  | 0.15 | 6.86 |  | 0.13 | 7.94 |  | 0.11 | 9.19 |
| Copper intake | Continuous variables | 0.57 | 1.75 |  | 0.51 | 1.95 |  | 0.64 | 1.56 |  | 0.58 | 1.74 |  | 0.18 | 5.55 |
| Manganese intake | Continuous variables | 0.51 | 1.97 |  | 0.39 | 2.58 |  | 0.60 | 1.66 |  | 0.50 | 2.00 |  | 0.29 | 3.50 |
| Zinc intake | Continuous variables | 0.33 | 3.06 |  | 0.31 | 3.18 |  | 0.38 | 2.66 |  | 0.32 | 3.10 |  | 0.29 | 3.42 |

TOL: tolerance; VIF: variance inflation factor. The tolerance of all variables is greater than 0.1 and the variance inflation factor is less than 10, so there is no collinearity between the variables. There was collinearity among energy, protein, fat and carbohydrate, so the three macronutrients were not included as independent variables.

Supplementary Table 2. Baseline characteristics of participants among non-CVD and new-onset CVD

| Characteristics | Total | Non-CVD | New-onset CVD | *P*-value |
| --- | --- | --- | --- | --- |
| N | 11470 | 11039 | 431 |  |
| Male, no. (%) | 5349 (46.63) | 5116 (46.34) | 233 (54.06) | 0.0016 |
| Han race, no. (%) | 10215 (89.06) | 9834 (89.08) | 381 (88.40) | 0.6549 |
| Urban, no. (%) | 4464 (38.92) | 4302 (38.97) | 162 (37.59) | 0.5632 |
| Smoking, no. (%) | 3644 (31.77) | 3471 (31.44) | 173 (40.14) | 0.0001 |
| Drinking, no. (%) | 3818 (33.29) | 3670 (33.25) | 148 (34.34) | 0.6367 |
| Hypertension, no. (%) | 2873 (25.05) | 2623 (23.76) | 250 (58.00) | < 0.0001 |
| Diabetes, no. (%) | 253 (2.21) | 226 (2.05) | 27 (6.26) | < 0.0001 |
| Marital status, no. (%) |  |  |  |  |
| Never married | 688 (6.00) | 684 (6.20) | 4 (0.93) | < 0.0001 |
| Married | 9884 (86.17) | 9516 (86.20) | 368 (85.38) |  |
| Divorced, separated, widowed, etc | 898 (7.83) | 839 (7.60) | 59 (13.69) |  |
| Education level, no. (%) |  |  |  |  |
| ≤ Primary school | 4372 (38.12) | 4109 (37.22) | 263 (61.02) | < 0.0001 |
| Middle school | 3718 (32.41) | 3625 (32.84) | 93 (21.58) |  |
| ≥ High school | 3380 (29.47) | 3305 (29.94) | 75 (17.40) |  |
| Activity level, no. (%) |  |  |  |  |
| Light | 6148 (53.60) | 5898 (53.43) | 250 (58.00) | < 0.0001 |
| Middle | 1752 (15.27) | 1719 (15.57) | 33 (7.66) |  |
| Heavy | 3570 (31.12) | 3422 (31.00) | 148 (34.34) |  |
| Age (years) | 48.00 (37.00, 58.00) | 47 (37, 57) | 60.00 (52.00, 69.00) | < 0.0001 |
| BMI (kg/m^2^) | 23.02 (20.90, 25.46) | 22.98 (20.86, 25.39) | 24.35 (21.80, 27.14) | < 0.0001 |
| Energy (kcal/day) | 1388.2 (1071.17, 1774.62) | 1385.08 (1068.95, 1772.95) | 1468.02 (1154.43, 1867.07) | 0.0020 |
| Dietary fiber (g/day) | 8.67 (6.21, 11.98) | 8.65 (6.20, 11.95) | 9.49 (6.40, 13.16) | 0.0025 |
| Niacin (mg/day) | 11.11 (7.92, 15.07) | 11.12 (7.93, 15.07) | 10.91 (7.69, 15.30) | 0.8974 |
| Vitamin C (mg/day) | 64.87 (42.25, 94.50) | 64.64 (42.20, 94.38) | 68.75 (45.04, 101.65) | 0.0506 |
| Vitamin E (mg/day) | 8.02 (5.29, 12.00) | 7.98 (5.27, 11.95) | 9.02 (6.16, 13.13) | < 0.0001 |
| calcium (mg/day) | 287.89 (203.11, 408.78) | 287.85 (202.97, 408.53) | 290.14 (205.53, 414.80) | 0.6742 |
| Iron (mg/day) | 18.89 (15.07, 23.68) | 18.93 (15.09, 23.69) | 18.24 (14.66, 23.19) | 0.1422 |
| Selenium (mg/day) | 32.58 (23.21, 46.59) | 32.56 (23.18, 46.62) | 33.29 (23.89, 45.52) | 0.8659 |
| Magnesium (mg/day) | 221.26 (168.22, 285.59) | 220.6 (167.83, 284.52) | 233.08 (179.43, 303.40) | 0.0004 |
| Copper (mg/day) | 1.41 (1.04, 1.95) | 1.41 (1.04, 1.94) | 1.5 (1.11, 1.98) | 0.0171 |
| Manganese (mg/day) | 4.22 (3.12, 5.62) | 4.21 (3.11, 5.59) | 4.72 (3.52, 6.22) | < 0.0001 |
| Zinc (mg/day) | 10.49 (8.35, 12.86) | 10.51 (8.37, 12.88) | 9.94 (8.01, 12.42) | 0.0087 |

Median (q1, q3) for continuous variable and numbers (percentage) for categorical variables. BMI: body mass index.

**Supplementary Table 3.** Association between zinc intake from different sources and risk of CVD

|  |  | Zinc intake (mg/day) | | |  | *P*-value for |
| --- | --- | --- | --- | --- | --- | --- |
| CVD | Q1 | Q2 | Q3 | Q4 | Q5 | trend |
| Total zinc intake (mg/day) | (< 7.87) | (7.87–9.63) | (9.63–11.38) | (11.38–13.56) | (≥ 13.56) |  |
| Cases | 102 | 97 | 82 | 71 | 79 |  |
| Incidence density | 8.56 | 6.17 | 4.82 | 4.12 | 5.06 |  |
| Model 1 HR (95%CI) | 1.00 | 0.71 (0.54, 0.93) | 0.55 (0.41, 0.74) | 0.47 (0.35, 0.64) | 0.58 (0.43, 0.78) | < 0.0001 |
| Model 2 HR (95%CI) | 1.00 | 0.72 (0.54, 0.96) | 0.58 (0.42, 0.78) | 0.48 (0.34, 0.67) | 0.39 (0.26, 0.58) | < 0.0001 |
| Model 3 HR (95%CI) | 1.00 | 0.73 (0.55, 0.97) | 0.57 (0.42, 0.78) | 0.47 (0.34, 0.66) | 0.40 (0.27, 0.60) | < 0.0001 |
| Model 4 HR (95%CI) | 1.00 | 0.72 (0.54, 0.97) | 0.59 (0.42, 0.81) | 0.50 (0.34, 0.72) | 0.44 (0.27, 0.71) | 0.0026 |
| Zinc intake from meat (mg/day) | (< 1.02) | (1.02–1.79) | (1.79–2.63) | (2.63–3.81) | (≥ 3.81) |  |
| Cases | 126 | 94 | 79 | 64 | 68 |  |
| Incidence density | 8.03 | 5.76 | 4.94 | 4.01 | 5.02 |  |
| Model 1 HR (95%CI) | 1.00 | 0.722 (0.55, 0.94) | 0.62 (0.47, 0.82) | 0.50 (0.37, 0.68) | 0.63 (0.47, 0.85) | < 0.0001 |
| Model 2 HR (95%CI) | 1.00 | 0.725 (0.56, 0.95) | 0.63 (0.48, 0.84) | 0.56 (0.41, 0.75) | 0.67 (0.50, 0.92) | 0.0008 |
| Model 3 HR (95%CI) | 1.00 | 0.673 (0.51, 0.88) | 0.57 (0.43, 0.76) | 0.52 (0.38, 0.71) | 0.61 (0.44, 0.85) | 0.0001 |
| Model 4 HR (95%CI) | 1.00 | 0.693 (0.53, 0.91) | 0.58 (0.43, 0.79) | 0.53 (0.38, 0.75) | 0.63 (0.43, 0.91) | 0.0012 |
| Zinc intake from other sources (mg/day) | (< 5.90) | 5.90–7.34) | (7.34–8.62) | (8.62–10.41) | (≥ 10.41) |  |
| Cases | 83 | 93 | 96 | 71 | 88 |  |
| Incidence density | 8.13 | 5.91 | 5.59 | 4.02 | 5.27 |  |
| Model 1 HR (95%CI) | 1.00 | 0.70 (0.52, 0.94) | 0.66 (0.49, 0.88) | 0.47 (0.34, 0.65) | 0.62 (0.46, 0.84) | 0.0002 |
| Model 2 HR (95%CI) | 1.00 | 0.76 (0.56, 1.02) | 0.75 (0.55, 1.02) | 0.51 (0.36, 0.73) | 0.49 (0.34, 0.72) | 0.0008 |
| Model 3 HR (95%CI) | 1.00 | 0.71 (0.52, 0.96) | 0.68 (0.50, 0.93) | 0.46 (0.33, 0.66) | 0.47 (0.32, 0.69) | 0.0002 |
| Model 4 HR (95%CI) | 1.00 | 0.73 (0.53, 0.99) | 0.70 (0.50, 0.97) | 0.50 (0.35, 0.73) | 0.54 (0.35, 0.85) | 0.0105 |

**Model 1:** non-adjusted. **Model 2**: adjusted for age, gender, race, and energy. **Model 3:** adjusted for age, gender, race, energy, residence, marital status, education level, activity level, smoking status, drinking status, BMI, hypertension, and diabetes. **Model 4:** adjusted for age, gender, race, energy intake, residence, marital status, education level, activity level, smoking status, drinking status, BMI, hypertension, diabetes, dietary fiber, niacin, vitamin C, vitamin E, calcium, iron, selenium, magnesium, copper, and manganese. CVD: cardiovascular disease; HR: hazard ratio; Incidence density: 1/1000 person-year.

**Supplementary Table 4.** Sensitivity analyses of further adjustments for the intake of cereals, potatoes, vegetables, fruits, nuts, meat, poultry, fish and shrimp, milk and eggs

|  |  | Dietary zinc intake (mg/day) | | |  |  |
| --- | --- | --- | --- | --- | --- | --- |
| CVD | Q1 (< 7.87) | Q2 (7.87–9.63) | Q3 (9.63–11.38) | Q4 (11.38–13.56) | Q5 (≥13.56) | *P*-value for trend |
| Cases | 102 | 97 | 82 | 71 | 79 |  |
| Incidence density | 8.56 | 6.17 | 4.82 | 4.12 | 5.06 |  |
| HR (95%CI) | 1.00 | 0.75 (0.56, 1.01) | 0.66 (0.48, 0.92) | 0.58 (0.40, 0.85) | 0.55 (0.34, 0.88) | 0.0590 |

CVD: cardiovascular disease; HR: hazard ratio; Incidence density: 1/1000 person-year.

**Supplementary Table 5.** Sensitivity analyses with RNI as the cut-off point

|  | Dietary zinc intake (mg/day) | |  |
| --- | --- | --- | --- |
| CVD | < RNI | ≥ RNI | *P*-value |
| Cases | 209 | 222 |  |
| Incidence density | 7.30 | 4.55 |  |
| Model 1 HR (95%CI) | 1.00 | 0.62 (0.51, 0.75) | < 0.0001 |
| Model 2 HR (95%CI) | 1.00 | 0.66 (0.52, 0.84) | 0.0008 |
| Model 3 HR (95%CI) | 1.00 | 0.64 (0.51, 0.82) | 0.0004 |
| Model 4 HR (95%CI) | 1.00 | 0.71 (0.55, 0.93) | 0.0117 |

**Model 1:** non-adjusted. **Model 2**: adjusted for age, gender, race, and energy. **Model 3:** adjusted for age, gender, race, energy, residence, marital status, education level, activity level, smoking status, drinking status, BMI, hypertension, and diabetes. **Model 4:** adjusted for age, gender, race, energy intake, residence, marital status, education level, activity level, smoking status, drinking status, BMI, hypertension, diabetes, dietary fiber, niacin, vitamin C, vitamin E, calcium, iron, selenium, magnesium, copper, and manganese. CVD: cardiovascular disease; HR: hazard ratio; RNI: Recommended Nutrient Intake (12.50 mg/day for male and 7.50 mg/day for female aged 18 years and above); Incidence density: 1/1000 person-year.

**Supplementary Table 6.** Sensitivity analyses with EAR as the cut-off point

|  | Dietary zinc intake (mg/day) | |  |
| --- | --- | --- | --- |
| CVD | < EAR | ≥ EAR | *P*-value |
| Cases | 136 | 295 |  |
| Incidence density | 9.46 | 4.68 |  |
| Model 1 HR (95%CI) | 1.00 | 0.49 (0.40, 0.60) | < 0.0001 |
| Model 2 HR (95%CI) | 1.00 | 0.55 (0.43, 0.70) | < 0.0001 |
| Model 3 HR (95%CI) | 1.00 | 0.53 (0.43, 0.69) | < 0.0001 |
| Model 4 HR (95%CI) | 1.00 | 0.58 (0.45, 0.76) | < 0.0001 |

**Model 1:** non-adjusted. **Model 2**: adjusted for age, gender, race, and energy. **Model 3:** adjusted for age, gender, race, energy, residence, marital status, education level, activity level, smoking status, drinking status, BMI, hypertension, and diabetes. **Model 4:** adjusted for age, gender, race, energy intake, residence, marital status, education level, activity level, smoking status, drinking status, BMI, hypertension, diabetes, dietary fiber, niacin, vitamin C, vitamin E, calcium, iron, selenium, magnesium, copper, and manganese. CVD: cardiovascular disease; HR: hazard ratio; EAR: Estimated Average Requirement (10.40 mg/day for male and 6.10 mg/day for female aged 18 years and above); Incidence density: 1/1000 person-year.

**Supplementary Table 7.** Sensitivity analyses excluding diabetic patients

|  |  | Dietary zinc intake (mg/day) | | |  |  |
| --- | --- | --- | --- | --- | --- | --- |
| CVD | Q1 (< 7.90) | Q2 (7.90–9.65) | Q3 (9.65–11.40) | Q4 (11.40–13.57) | Q5 (≥13.57) | *P*-value for trend |
| Cases | 95 | 93 | 75 | 69 | 72 |  |
| Incidence density | 8.00 | 6.00 | 4.50 | 4.09 | 4.66 |  |
| Model 1 HR (95%CI) | 1.00 | 0.73 (0.55, 0.98) | 0.55 (0.40, 0.74) | 0.50 (0.36, 0.68) | 0.57 (0.42, 0.77) | < 0.0001 |
| Model 2 HR (95%CI) | 1.00 | 0.73 (0.55, 0.98) | 0.56 (0.41, 0.77) | 0.49 (0.35, 0.69) | 0.37 (0.24, 0.55) | < 0.0001 |
| Model 3 HR (95%CI) | 1.00 | 0.71 (0.53, 0.96) | 0.55 (0.40, 0.76) | 0.48 (0.34, 0.68) | 0.37 (0.24, 0.56) | < 0.0001 |
| Model 4 HR (95%CI) | 1.00 | 0.72 (0.53, 0.97) | 0.56 (0.40, 0.79) | 0.50 (0.34, 0.74) | 0.38 (0.23, 0.64) | 0.0021 |

**Model 1:** non-adjusted. **Model 2**: adjusted for age, gender, race, and energy. **Model 3:** adjusted for age, gender, race, energy, residence, marital status, education level, activity level, smoking status, drinking status, BMI, hypertension, and diabetes. **Model 4:** adjusted for age, gender, race, energy intake, residence, marital status, education level, activity level, smoking status, drinking status, BMI, hypertension, diabetes, dietary fiber, niacin, vitamin C, vitamin E, calcium, iron, selenium, magnesium, copper, and manganese. CVD: cardiovascular disease; HR: hazard ratio; Incidence density: 1/1000 person-year.

**Supplementary Table 8.** Sensitivity analysis of missing values was performed after multiple imputation

|  |  | Dietary zinc intake (mg/day) | | |  |  |
| --- | --- | --- | --- | --- | --- | --- |
| CVD | Q1 (< 7.80) | Q2 (7.80–9.58) | Q3 (9.58–11.35) | Q4 (11.35–13.56) | Q5 (≥13.56) | *P*-value for trend |
| Cases | 118 | 107 | 93 | 75 | 88 |  |
| Incidence density | 9.14 | 6.2 | 5.03 | 3.98 | 5.12 |  |
| Model 1 HR (95%CI) | 1.00 | 0.66 (0.51, 0.86) | 0.54 (0.41, 0.70) | 0.42 (0.32, 0.57) | 0.55 (0.41, 0.72) | < 0.0001 |
| Model 2 HR (95%CI) | 1.00 | 0.67 (0.51, 0.87) | 0.56 (0.42, 0.74) | 0.42 (0.31, 0.58) | 0.35 (0.24, 0.51) | < 0.0001 |
| Model 3 HR (95%CI) | 1.00 | 0.65 (0.49, 0.85) | 0.54 (0.40, 0.72) | 0.41 (0.30, 0.56) | 0.36 (0.25, 0.52) | < 0.0001 |
| Model 4 HR (95%CI) | 1.00 | 0.63 (0.48, 0.83) | 0.53 (0.39, 0.72) | 0.41 (0.29, 0.58) | 0.36 (0.23, 0.56) | < 0.0001 |

**Model 1:** non-adjusted. **Model 2**: adjusted for age, gender, race, and energy. **Model 3:** adjusted for age, gender, race, energy, residence, marital status, education level, activity level, smoking status, drinking status, BMI, hypertension, and diabetes. **Model 4:** adjusted for age, gender, race, energy intake, residence, marital status, education level, activity level, smoking status, drinking status, BMI, hypertension, diabetes, dietary fiber, niacin, vitamin C, vitamin E, calcium, iron, selenium, magnesium, copper, and manganese. CVD: cardiovascular disease; HR: hazard ratio; Incidence density: 1/1000 person-year.
